# Improvement of the Pacific bluefin tuna (*Thunnus orientalis*) reference genome and development of male-specific DNA markers

**DOI:** 10.1038/s41598-019-50978-4

**Published:** 2019-10-08

**Authors:** Ayako Suda, Issei Nishiki, Yuki Iwasaki, Aiko Matsuura, Tetsuya Akita, Nobuaki Suzuki, Atushi Fujiwara

**Affiliations:** 1 Research Center for Bioinformatics and Biosciences, National Research Institute of Fisheries Science, Japan Fisheries Research and Education Agency, Yokohama, 236-8648 Japan; 2 Research Center for Fisheries Resources, National Research Institute of Fisheries Science, Japan Fisheries Research and Education Agency, Yokohama, 236-8648 Japan; 3 Fisheries Agency, Ministry of Agriculture, Forestry and Fisheries, Tokyo, 100-8907 Japan; 4 National Research Institute of Far Seas Fisheries, Japan Fisheries Research and Education Agency, Shimizu, Shizuoka, 424-8633 Japan

**Keywords:** Genetic markers, Genome-wide association studies

## Abstract

The Pacific bluefin tuna, *Thunnus orientalis*, is a highly migratory species that is widely distributed in the North Pacific Ocean. Like other marine species, *T. orientalis* has no external sexual dimorphism; thus, identifying sex-specific variants from whole genome sequence data is a useful approach to develop an effective sex identification method. Here, we report an improved draft genome of *T. orientalis* and male-specific DNA markers. Combining PacBio long reads and Illumina short reads sufficiently improved genome assembly, with a 38-fold increase in scaffold contiguity (to 444 scaffolds) compared to the first published draft genome. Through analysing re-sequence data of 15 males and 16 females, 250 male-specific SNPs were identified from more than 30 million polymorphisms. All male-specific variants were male-heterozygous, suggesting that *T. orientalis* has a male heterogametic sex-determination system. The largest linkage disequilibrium block (3,174 bp on scaffold_064) contained 51 male-specific variants. PCR primers and a PCR-based sex identification assay were developed using these male-specific variants. The sex of 115 individuals (56 males and 59 females; sex was diagnosed by visual examination of the gonads) was identified with high accuracy using the assay. This easy, accurate, and practical technique facilitates the control of sex ratios in tuna farms. Furthermore, this method could be used to estimate the sex ratio and/or the sex-specific growth rate of natural populations.

## Introduction

The Pacific bluefin tuna, *Thunnus orientalis* is widely distributed in the North Pacific Ocean, and is one of the most important commercial fishes globally. Because of the high demand for this species and its high market price, the targeted fishing of species in the tuna genus has led to overfishing and drastic population declines^1,2^. Mature fishes have high market value, with juveniles also being targeted by the current market and aquaculture^3^. Japan, like many other countries, uses tuna products; thus, it is important to monitor this resource to ensure it is managed effectively. However, at present, management advice is based on the results of stock assessments using simple population models, such as non-spatial structure and single-sex models, due to the lack of biological information^3^. Furthermore, the development of full-life-cycle aquaculture systems that operate efficiently represents one of the countermeasures in place to conserve natural resources^4,5^.

Japan began developing the full-life-cycle aquaculture system for Pacific bluefin tuna in the 1970s, and succeeded in 2002^4,5^. Knowledge about culturing tuna has accrued, with many studies being dedicated towards understanding the optimum rearing conditions for this species; however, a number of issues remain unresolved^6^. One of issues in full-life-cycle aquaculture is unstable egg collection^6,7^, because spawning is strongly influenced by environmental factors, such as water temperature^6,7^. Consequently, the maturation of the ovaries is subject to variation, with only some females spawning under aquaculture conditions, reducing genetic diversity^6,8^. Moreover, females often die after the spawning season under cultured conditions, increasing the ratio of males in sea cages. Although controlling the sex ratio in a sea cage to have a bias to females might increase the production of fertilised eggs^9^, the Pacific bluefin tuna lacks morphological sexual dimorphism, making it difficult to identify and remove males through visual inspections. Sex is often distinguished by observing the gonads; however, this operation is lethal and inconclusive in juvenile fish. Thus, effective, simple, and accurate sex identification methods that can be tested on juveniles are required in tuna farming. Sex identification, especially in juveniles, is a reasonable strategy for controlling the sex ratio before individuals are transferred from indoor tanks to open sea net cages, as it would reduce operational costs. Therefore, the development of a sex identification method using molecular markers could be potentially used to resolve current difficulties with the aquaculture of this species.

Identifying the genetic sex of an individual using several types of molecular markers has been achieved in various marine species. For example, two male-specific DNA markers have been developed in African catfish, *Clarias gariepinus*^10^ using the random amplified polymorphic DNA (RAPD) technique. Amplified fragment length polymorphism (AFLP) has been used to identify seven female-specific markers in half-smooth tongue sole, *Cynoglossus semilaevis*^11^, while three male-specific markers have been developed in Nile tilapia, *Oreochromis niloticus*^12^. Linkage analysis using the bacterial artificial chromosome library or microsatellite markers have facilitated the discovery of various sex determination (SD) regions/genes in teleosts, including *dmY*^13,14^, *sox3*^15^, and *gsdfY*^16^ in the medaka groups, *amhr2* in the fugu groups^17–19^, and a candidate gene in yellowtail^20–22^. With the advent of sequencing techniques^23^, whole genome sequence data have provided new insights on the SD system of Nile tilapia^24^, half-smooth tongue sole^25^, Killifish^26^, and Atlantic cod^27^. However, sex-specific markers and SD genes have not yet been identified in Pacific bluefin tuna. One previous study described a male-specific DNA sequence in broodstock Pacific bluefin tuna, *Male delta 6* (*Md6*)^9^, using AFLP-selective DNA amplification products, followed by high-throughput sequencing. Although *Md6* identifies sex with relatively high accuracy (accuracy: 84% in F2 and 94% in F3) in broodstock fish, its accuracy is low in wild individuals (accuracy: 39%). Thus, there is a need to develop sex-specific markers that are highly accurate for both aquaculture and wild individuals.

Here, hybrid assembly with a combination of Illumina sequence data and PacBio long molecule sequence reads was applied to construct an improved draft genome of the Pacific bluefin tuna. Subsequently, we analysed re-sequencing data from 15 males and 16 females to investigate sex-specific regions through genome-wide association studies (GWAS) using the improved draft genome. PCR primers and a PCR-based sex identification assay were developed using male-specific variants. We also evaluated the accuracy of sex identification using the assay on 115 individuals (56 males and 59 females). Our results are expected to demonstrate whether highly accurate sex-specific DNA markers could be used to identify the genetic sex of individuals, potentially representing a practical technique to control sex ratio in tuna farming. This sex identification assay could also be used to identify the sex ratio and/or the sex-specific growth rate of juveniles in natural populations.

## Results

### Construction of an improved draft genome

Overall, 18.3 Gb sequence data (×215.4 coverage) from Illumina and 1.85 Gb (×21.8 coverage) from PacBio were obtained (Supplementary Table S1). Supplementary Table S2 and Supplementary Fig. S1 show the total reads used in the assembling processes and the assembly pipeline, respectively. The sequence data were subject to seven assembling steps (Supplementary Fig. S1, Supplementary Table S3). As a result, the final genome assembly was 787 Mb, with a scaffold N50 size of 7.92 Mb with 444 scaffolds (Table 1 and Supplementary Table S3). This draft genome is substantially improved compared with previously reported draft genome of the Pacific bluefin tuna^28^. There was a 38-fold increase in scaffold contiguity, a 40-fold increase in average scaffold length, a 58-fold increase in the N50 scaffold, and a 148-fold reduction in the number of gaps (Table 1). In the tuna draft genome, the presence of 225 out of the 233 (96.57%) CEGMA genes was confirmed, and 222 out of the 225 genes were complete core genes (95.28%). BUSCO results indicated that 2,303 genes (89.0%) were complete BUSCOs. This result demonstrates the completeness and high quality of the present assembly (Table 1).

**Table 1:** Comparison of genome assembly and completeness assessment results between the previous genome (Tuna_1^28^) and new genome assembled in this study (Tuna_2) using CEGMA and BUSCO.

Assembly Statistics	Tuna_1	Tuna_2
**Contig statistics**		
Number of contigs	135,841	1,248
Total contig size (bp)	684,478,122	786,014,188
Contig N50 size (bp)	8,173	3,075,225
Largest contig (bp)	79,059	14,489,242
**Scaffold statistics**		
Number of scaffolds	16,801	444
Total scaffold size (bp)	740,348,846	786,596,543
Scaffold N50 size (bp)	136,950	7,922,002
Largest scaffold size (bp)	1,021,118	19,788,065
GC content (%)	40	40
Number of gaps	119,041	804
**Completeness Assessment Results Using CEGMA**		
Total number of core genes queried	233	233
**Number of core genes detected**		
Complete	176 (75.54%)	222 (95.28%)
Complete + Partial	222 (95.28%)	225 (96.57%)
Number of missing core genes	11 (4.72%)	8 (3.43%)
Average number of orthologs per core genes	1.38	1.03
% of detected core genes that have more than 1 ortholog	26.14	3.15
**Completeness Assessment Results using BUSCO**		
Total BUSCO groups searched	4584	4584
Complete BUSCOs	4021 (87.7%)	4044 (88.2%)
Complete and single-copy BUSCOs	3933 (85.8%)	3848 (83.9%)
Complete and duplicated BUSCOs	88 (1.9%)	196 (4.3%)
Fragmented BUSCOs	337 (7.4%)	222 (4.8%)
Missing BUSCOs	226 (4.9%)	318 (7.0%)

### Identification of male-specific variants

**Figure 1:**
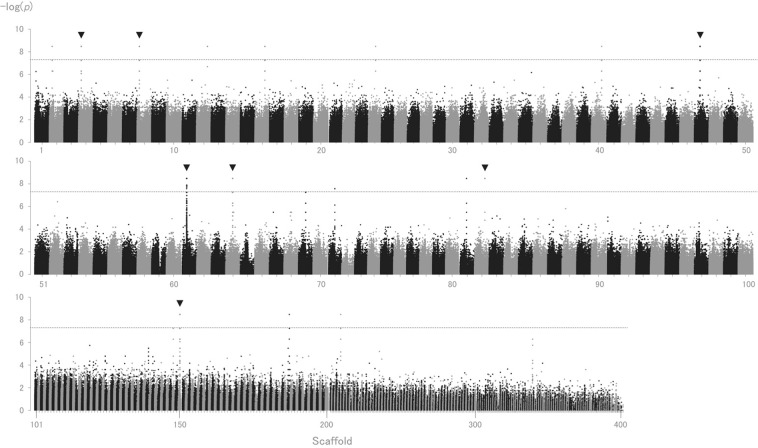
Manhattan plot of sex-specific genotypes in Pacific bluefin tuna (*Thunnus orientalis)*. Exact *p*-values using Fisher’s exact test in the 2-by-3 table of genotypes were -log transformed and are represented on the y-axis, along with scaffold number from one to 444 on the horizontal axis. Seven distinct regions, shown by arrows, attained the genome-wide significance threshold (*p* = 5 × 10^−8^), containing more than 10 male-specific SNPs in each scaffold. Dashed line represents the genome-wide significance threshold. Scaffold 101 to 444 are compressed for visualisation.

Thirty-one individual (15 males and 16 females) sequence datasets were obtained from NextSeq. 500, with an average sequence coverage of 25× per individual (Supplementary Table S4). GWAS was conducted to detect biallelic SNPs that show strict sex-specific segregation (i.e. genotypes either exclusively homozygous or heterozygous depending on sex) by aligning the 31 individual sequence datasets to an improved draft genome. A total of 30,116,708 biallelic SNPs were retained, of which 250 SNPs showed sex-specific segregation in 16 scaffolds, with *p*-values achieving the genome-wide significance threshold (*p*-values < 5 × 10^−8^) (Fig. 1, Supplementary Table S5). All sex-specific SNPs displayed male-heterozygous segregation patterns (hereafter, referred to as “male-specific SNPs”).

**Figure 2:**
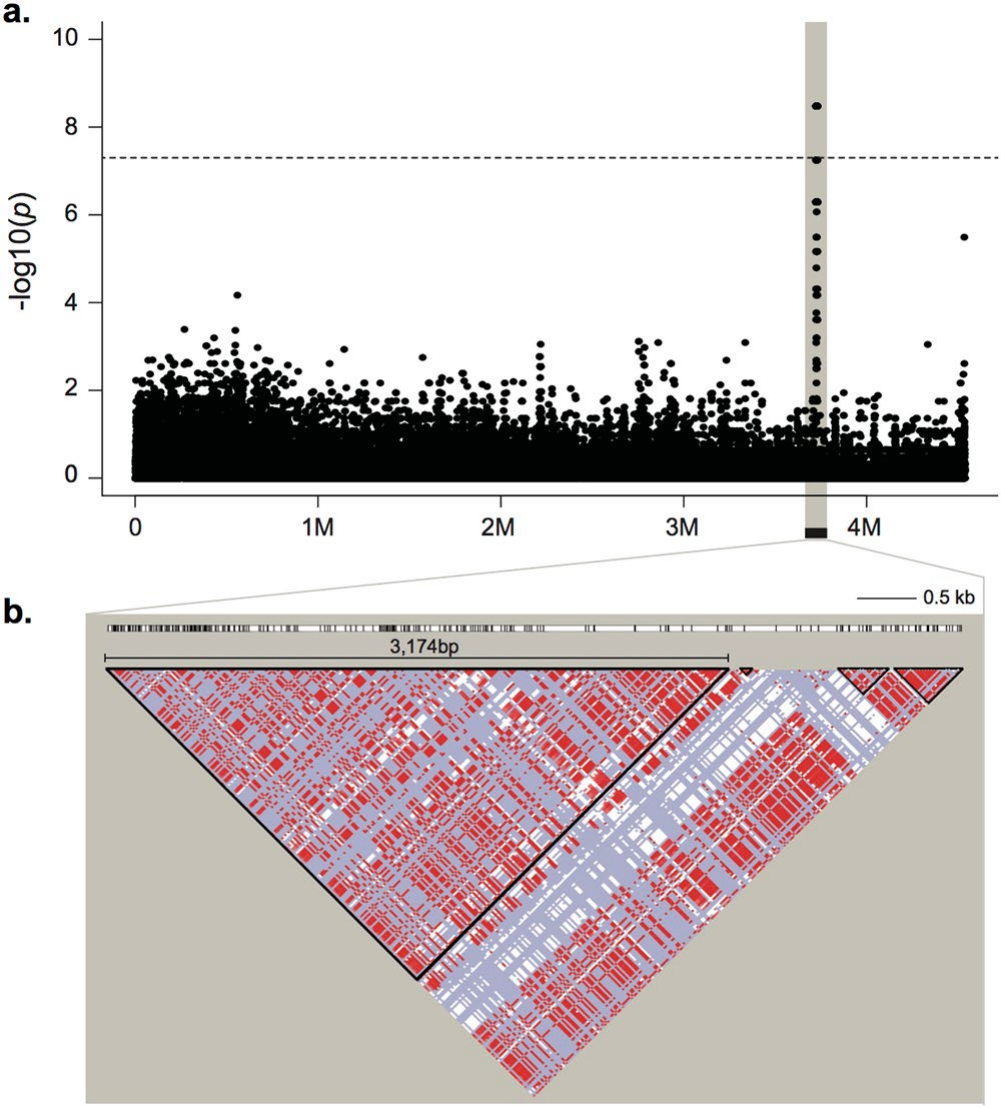
Manhattan plot and linkage disequilibrium (LD) plot of sex-specific polymorphisms in scaffold_064. (**a**) The polymorphisms in the shaded region include variant sites that attained the genome-wide significance threshold (*p*-values < 5 × 10^−8^), approximately 6.5 kb. (**b**) LD block was determined by Haploview 4.2, with an LD-based partitioning algorithm. The colour scheme of the logarithm of the odds (LOD) score and D’ indicate: red: D’ = 1 and LOD ≥ 2; blue: D’ = 1 and LOD < 2; and white: D’ < 1 and LOD < 2. The largest LD block is 3,174 bp.

**Figure 3:**
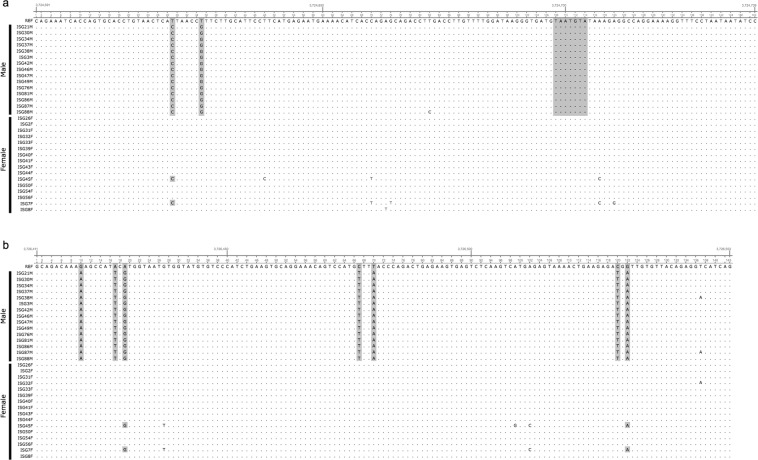
Nucleotide sequence of sex-specific regions in scaffold_064 that was targeted for sex identification amplification. Fifteen male and 16 female sequences are aligned, and sex-specific genotypes are shown in the shaded sections. (**a**) 113 bp of male-specific products, 142 bp and 149 bp products are amplified for male-specific products and both sexes, respectively. (**b**) 143 bp of male-specific products are targeted to amplify males-specific products. Note, each allele is detected as heterozygous genotypes; hence, alternatives are the same as the reference.

Seven out of the 16 scaffolds contained more than 10 male-specific SNPs (Fig. 1.). The LD analyses revealed that the largest LD block was on scaffold_064 (approximately 4.5 Mb in scaffold size). Scaffold_064 contains 44 male-specific SNPs within an approximately 6.5 kb region, where four LD blocks were present (Table 2). The largest LD block, 3,174 bp, was within this region (Fig. 2). The LD block contained the largest number of male-specific SNPs is the region where recombination is extensively suppressed. Thus, this region might be important for sex determination and to investigate male-specific markers. Further variant detection using Haplotypecaller in GATK was performed on scaffold_064. Seven additional male-specific variants (SNPs and one indel) were detected in the same region, 6.5 kb (position 3723782–3730295) by performing Unifiedgenotyper analysis. All male-specific variants displayed male-heterozygous (Fig. 3, Table 3).

**Table 2:** Scaffolds and linkage disequilibrium for scaffolds containing male-specific SNPs.

Scaffold	Scaffold Size (bp)	Number of Total SNPs	Number of Sex-specific SNPs	Start (bp)	End (bp)	Size (bp)	Number of LD block	Maximum LD block size (bp)	LD Start (bp)	LD End (bp)
Scaffold_002	19,343,486	634,171	2	1411525	1411537	13	—	—	—	—
**Scaffold_004**	**15,333,484**	**729,773**	**58**	**13755422**	**13757959**	**2538**	**21**	**406**	**13757866**	**13758271**
**Scaffold_008**	**13,582,522**	**414,429**	**18**	**13181867**	**13182926**	**1060**	**6**	**71**	**13182469**	**13182539**
Scaffold_012	11,623,986	535,318	4	6658078	6658121	44	—	—	—	—
Scaffold_016	10,992,500	349,783	4	5851008	5851271	264	—	—	—	—
Scaffold_024	9,474,074	378,040	2	4640584	4641391	808	—	—	—	—
Scaffold_040	6,874,678	235,900	1	6336490	6336490	1	—	—	—	—
**Scaffold_047**	**5,831,790**	**228,235**	**32**	**2802518**	**2810509**	**7992**	**23**	**208**	**2810382**	**2810589**
**Scaffold_061**	**4,681,469**	**154,850**	**29**	**1942591**	**2033284**	**90694**	**206**	**307**	**2026362**	**2026668**
**Scaffold_064**	**4,535,926**	**89,006**	**44**	**3723782**	**3730295**	**6514**	**4**	**3,174**	**3723703**	**3726876**
Scaffold_081	3,464,053	53,509	2	3407626	3407890	265	—	—	—	—
**Scaffold_082**	**3,452,009**	**163,342**	**11**	**620729**	**620873**	**145**	**1**	**185**	**620689**	**620873**
**Scaffold_150**	**749,403**	**5,889**	**41**	**54606**	**58731**	**4126**	**19**	**1,204**	**56072**	**57275**
Scaffold_187	294,664	3,161	1	6162	6162	1	—	—	—	—
Scaffold_208	153,918	1,173	1	139056	139056	1	—	—	—	—

Number of total SNPs and sex-specific SNPs (*p* < 5 × 10^−8^) for each scaffold were extracted by comparing 31 individuals. For scaffolds that contain more than 10 sex-specific SNPs (in bold), the maximum linkage disequilibrium (LD) block was analyzed using PLINK v. 1.90b4.2 and Haploview 4.2. SNP calling was conducted by aligning to an improved draft genome using Genome Analysis Toolkit v. 3.6.

**Table 3:** Male-specific variants in scaffold_064.

Position (bp)	Allele		Genotype (Ref/Het/Alt)		*p*-value
	Reference	Alternative	Females	Males	
3723782	A	T	16/0/0	0/15/0	3.33E-09
3723876	A	G	16/0/0	0/15/0	3.33E-09
3723925	T	C	16/0/0	0/15/0	3.33E-09
3724050	C	T	16/0/0	0/15/0	3.33E-09
3724051	A	G	16/0/0	0/15/0	3.33E-09
3724070	A	G	16/0/0	0/15/0	3.33E-09
3724479	G	T	16/0/0	0/15/0	3.33E-09
3724481	T	G	16/0/0	0/15/0	3.33E-09
3724494	C	T	16/0/0	0/15/0	3.33E-09
3724556	C	T	16/0/0	0/15/0	3.33E-09
3724561	C	A	16/0/0	0/15/0	3.33E-09
3724625	T	G	16/0/0	0/15/0	3.33E-09
3724697	GTAATGTA	G	16/0/0	0/15/0	3.33E-09
3725864	T	C	16/0/0	0/15/0	3.33E-09
3725869	G	A	16/0/0	0/15/0	3.33E-09
3725870	C	T	16/0/0	0/15/0	3.33E-09
3725892	C	T	16/0/0	0/15/0	3.33E-09
3725903	G	A	16/0/0	0/15/0	3.33E-09
3725915	G	A	16/0/0	0/15/0	3.33E-09
3725924	G	C	16/0/0	0/15/0	3.33E-09
3725968	G	A	16/0/0	0/15/0	3.33E-09
3725974	G	A	16/0/0	0/15/0	3.33E-09
3726082	A	G	16/0/0	0/15/0	3.33E-09
3726098	C	T	16/0/0	0/15/0	3.33E-09
3726136	G	T	16/0/0	0/15/0	3.33E-09
3726155	A	G	16/0/0	0/15/0	3.33E-09
3726182	G	T	16/0/0	0/15/0	3.33E-09
3726338	G	C	16/0/0	0/15/0	3.33E-09
3726374	T	C	16/0/0	0/15/0	3.33E-09
3726420	G	A	16/0/0	0/15/0	3.33E-09
3726427	A	T	16/0/0	0/15/0	3.33E-09
3726477	C	T	16/0/0	0/15/0	3.33E-09
3726480	T	A	16/0/0	0/15/0	3.33E-09
3726530	C	T	16/0/0	0/15/0	3.33E-09
3726649	G	T	16/0/0	0/15/0	3.33E-09
3726723	T	A	16/0/0	0/14/0	6.88E-09
3726751	A	G	16/0/0	0/15/0	3.33E-09
3726760	C	T	16/0/0	0/15/0	3.33E-09
3726826	C	T	16/0/0	0/15/0	3.33E-09
3727016	C	T	16/0/0	0/15/0	3.33E-09
3727037	A	G	16/0/0	0/15/0	3.33E-09
3727050	C	T	16/0/0	0/15/0	3.33E-09
3727069	C	T	16/0/0	0/15/0	3.33E-09
3729537	C	A	16/0/0	0/15/0	3.33E-09
3729736	C	T	16/0/0	0/15/0	3.33E-09
3729866	T	C	16/0/0	0/15/0	3.33E-09
3730062	C	A	16/0/0	0/15/0	3.33E-09
3730221	C	T	16/0/0	0/15/0	3.33E-09
3730222	G	A	16/0/0	0/15/0	3.33E-09
3730268	C	T	16/0/0	0/15/0	3.33E-09
3730295	T	A	16/0/0	0/15/0	3.33E-09

All polymorphisms have homozygous in all females (n = 16) and heterozygous in all males (n = 14 or 15), where *p-*values are significant using Fisher’s exact test.

Ref; homozygous reference (Tuna_2), Het; heterozygous, Alt; homozygous alternative.

### Development of PCR-based sex identification method

**Figure 4:**
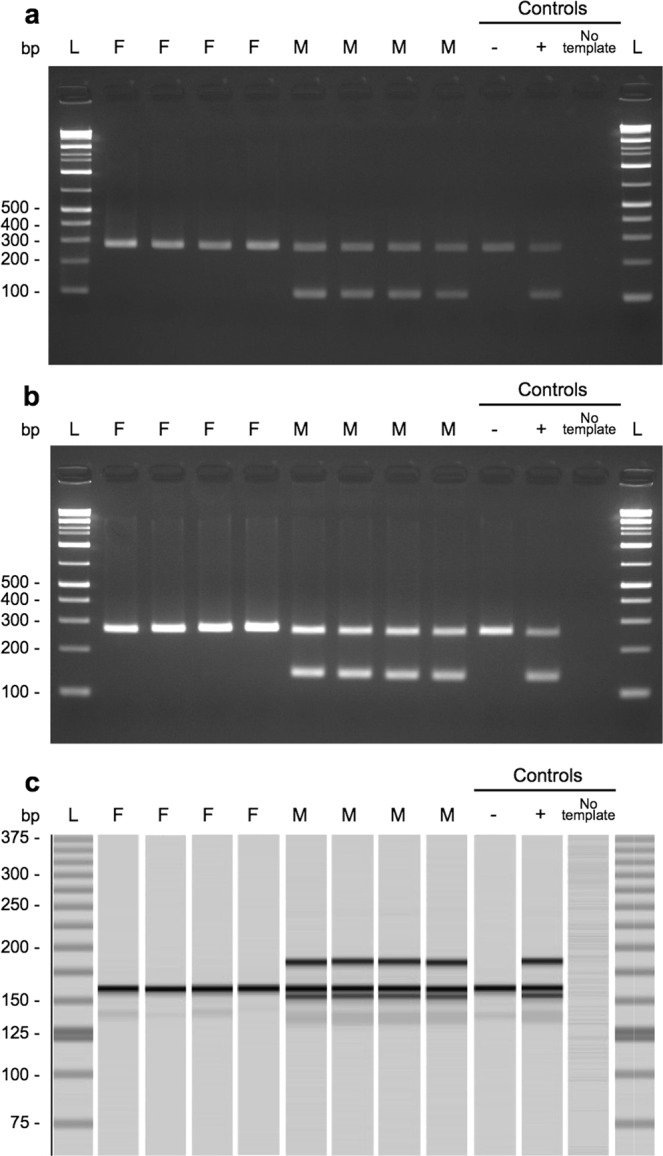
Sex-specific PCR products. 113 bp (**a**), 143 bp (**b**), and double-band products (**c**) were only present in males based on PCR amplification. ND4 products (268 bp) and internal controls were concurrently amplified with the same PCR conditions to avoid misclassification (a and b). Note, an approximately 180 bp band in c is a non-targeted product, but appeared to be male-specific amplification. Positive (+; sequenced male), negative (−; sequenced female), and no template added controls are shown on the right. All gel images were cropped and expanded for visualisation. Full-length gel images are presented in Supplementary Fig. 2.

PCR primers were designed within the largest LD block of scaffold_064 (Table 4, Fig. 3). Using primer pairs I and II, PCR demonstrated that all 15 males produced the male-specific band of 113 bp and 143 bp, respectively, but not all 16 females (Fig. 4a,b). Using primer pair III, a male-specific product (142 bp) and a product of both sexes (149 bp) were amplified (Fig. 4c). An extra band appeared, of which approximately 180 bp was a non-targeted product, but appeared to be male-specific amplification. Sex identification using this assay was tested on an additional 115 individuals (56 males and 59 females). PCR amplification using primer pair III identified sex with 100% accuracy. In comparison, PCR amplification using primer pairs I and II resulted in male-specific bands being observed or amplification failure in eight females (Supplementary Table 6).

**Table 4:** PCR primers for sex identification and an internal control, NADH dehydrogenase subunit 4 (ND4) gene.

Primer pair	Name	Sequence (5′ to 3′)	Size (bp)
Pair I	sca64_3724604_F	TGCACCTGTAACTCACTAACCG	113
	sca64_3724604_R	CCTTTTCCTGGCCTCTTTACAT	
Pair II	sca64_3726411_F	GCAGACAAAAAGCCATTCG	143
	sca64_3726411_R_A^*^	CTGATGACCTCTGTAACACAATCAT	
	sca64_3726411_R_T^*^	CTGATGTCCTCTGTAACACAATCAT	
Pair III	sca64_3724591_F	CAGAAATCACCAGTGCACC	142 and 149
	sca64_3724591_R	GGATATTATTAGGAAACCTTTTCCTG	
Pair IV	ND4_F	ACAGACCCGTTGTCAACTCC	268
	ND4_R	TCCCTGCATTTAAACGCTCT	

^*^primers were combined at equal concentrations.

### Gene annotation of the seven sex-specific regions

Gene annotation of an approximately 200 kb region surrounding the male-specific variants on the seven scaffolds was performed after gene predictions (Supplementary Table S7). Fifty-seven out of 183 predicted genes matched the protein sequences of *Oryzias latipes*. Two predicted genes were located on the largest LD block (3,174 bp). We confirmed that the sequences of these two predicted genes and four adjacent predicted genes were partially homologous to “ENSORLG00000010797”. This gene encodes “ATP-dependent DNA helicase” and its functions, explained by GO terms, are ‘telomere maintenance’, ‘DNA repair’, ‘cellular response to DNA damage stimulus’, ‘DNA recombination’, and ‘DNA duplex unwinding’ in biological processes (Supplementary Table S7). None of the regions matched the previously discovered SD genes or their associated genes (Supplementary Table S8).

## Discussion

Here we report the construction of an improved draft genome of the Pacific bluefin tuna, *Thunnus orientalis*, and the development of male-specific DNA markers to distinguish sex. Our sex identification method using male-specific variants is a PCR-based technique that is easy to perform, with highly accurate detection. Our sex identification assay could be of great practical use for controlling sex ratios under aquaculture conditions. It could also be used to obtain data from wild populations, providing useful information for the management and conservation of these natural stocks.

Following the publication of the Pacific bluefin tuna genome in 2013^29^, it has been widely referred to for transcriptomic comparison among *Thunnus* speceis^30^. This use included the development of a 44 K oligonucleotide microarray for Pacific bluefin tuna^31^, 15 K for Atlantic bluefin tuna^30^, and linkage map construction^32^. Despite being used as a reference, the previous draft genome is highly fragmented, with a large number of gaps in the scaffold, complicating analyses^33^. The completeness assessment on the improved draft genome supported the completeness and high quality of the present assembly when compared to the previous assembly (Table 1). In addition, our assembly has a larger fraction of the BUSCOs and CEGMA genes than the previously published draft genomes analysed in 66 teleost species^34^, supporting the completeness of the genome. Performing improved genome assemblies enhanced the integrity of the genome, eliminating many gaps and facilitating the acquisition of longer sequences when compared to the previous genome. As in other species, combining PacBio long reads with Illumina short reads sufficiently improves genome assemblies^35–38^. Our improved draft genome provides a solid foundation for future population and resource management studies of Pacific bluefin tuna.

When performing GWAS using resequencing data, 250 SNPs were male-heterozygous, with seven distinct regions being identified (Fig. 2 and Supplementary Table S5). The LD block pattern of these seven distinct regions showed small to large patterns with substantial signatures of recombination suppression (Table 2). All male-specific variants were male-heterozygous, suggesting that Pacific bluefin tuna have a male heterogametic sex-determination system. Assembly errors, or structural variation, such as a large insertion or deletion, might be causal for identifying multiple sex-specific regions across different scaffolds, because SD genes or SD regions are often detected as single gene or single region^39^. For example, in the medaka groups, *dmY*^13,14^, *sox3*^15^ and *gsdfY*^16^ are introduced as master SD genes, while *amhr2* is introduced to the fugu groups^17–19^. A single region is introduced to LG13 for Atlantic halibut^40^, and a single region is introduced to scaffold 22 for the California Yellowtail^41^. However, the detection of sex-specific SNPs across different scaffolds has also been confirmed in two rockfish species^42^ and Atlantic cod^27^. Differences in analyses, sample size, and type (whether family or wild specimens) might influence the different detection patterns of sex-associated regions^43^. On-going studies are now investigating chromosome level assembly, which is expected to provide a high-quality reference genome sequence with high sequence contiguity, accuracy, and improved gene annotation. This assembly could reveal the relationship among multiple sex-specific regions across different scaffolds. For instance, the sex-specific regions might converge into single chromosome.

Fifty-seven predicted genes were observed in an approximately 200 kb region surrounding each male-specific region according to BLASTP. One of the male-specific regions with the largest LD block (3,174 bp) on scaffold_064 was confirmed to contain homologous sequence, “ENSORLG00000010797” of which the GO term is “ATP dependent DNA helicase” in its biological process (Supplementary Table S7). The function of this gene is not known for *O. latipes* or other organisms. Thus, it is not possible to evaluate its sex-associated function at present. However, interestingly, the transcriptome analysis between females and males in *O. latipes* showed “ENSORLG00000010797” as one of the genes detected as an up-regulated gene in females^44^. Thus, its homologous sequence in Pacific bluefin tuna might be associated with its sex-specific function. Further studies are required, such as determining the complete sequences of this DNA helicase-like gene and comparing expression levels between males and females in future studies. A previously introduced male-specific sequence, *Md6*^9^, was detected in multiple regions on scaffold_101, scaffold_061, and scaffold_286 of our draft genome (data not shown), though the location of *Md6* was out of the range of any male-specific regions. In addition, the 6-bp deletion was not observed from our male data, thus *Md6* might be inherited from male parents to male progeny under aquaculture conditions, as suggested by Agawa *et al*.^9^.

By focusing on male-specific variants in the largest LD block (3,174 bp on scaffold_064), we were able to design PCR primers and a highly accurate assay to identify the sex of Pacific bluefin tuna (Supplementary Table 6). Sex identification using the PCR assay is expected to help improve tuna aquaculture techniques in the future. In aquaculture, sex detection is limited to adults, as it is usually conducted by directly observing gonads or histological examination. In comparison, sex identification using our PCR assay is easy, requiring minimal handling of individuals. Moreover, sex of juveniles can be identified using our method, allowing the sex ratio in cages to be adjusted at an early stage, which could enhance breeding programs. In our preliminary experiment, we confirmed that body mucus could be successfully used to identify sex with our method. This approach might be less stressful to tuna and requires less effort than sampling approaches based on fin clips or muscle tissues from live individuals.

Our sex-identification assay could be a valuable tool for understanding the biological characteristics of Pacific bluefin tuna. For example, it could be used to observe differences in the sex ratio of natural populations with respect to their spatio-temporal distribution. Although the sex ratio of the wild tuna population tends to be 1:1^45,46^, the sex ratio of juvenile fish is not known, or whether there are differences in the distribution between the sexes. Thus, our assay could be implemented in surveys that evaluate sex ratio analyses, rather than waiting for sexual maturity. Tracking the migration patterns of males and females using electronic tags also provides valuable information for the management of wild tuna fisheries. In addition, identifying the sex of other tuna species is an on-going process and depends on whether primers (pair I, II, and III) are available for cross-species amplification.

Several tuna species, such as the Atlantic bluefin tuna^47^, southern bluefin tuna^48,49^, bigeye tuna^50^, and albacore^51^, exhibit size dimorphism between the sexes, with males growing larger than females after reaching maturity. In an assessment of albacore stock, sex-specific growth curves are currently used^28^. In comparison, assessments of Pacific bluefin tuna estimate the stock using single–sex growth curves^3^, even though sexual size dimorphism between males and females seems to occur in older age groups^52,53^. To provide evidence of sexual size dimorphism, our assay could be used to identify the sex of wild populations and incorporate sex information into morphological characteristics. This approach would facilitate the creation of an appropriate growth curve for the two sexes. Such fundamental information is essential to estimate annual recruitment and to conduct realistic stock assessments.

## Methods

### Ethics statement

All animal handling and methods were carried out in accordance with the Guidelines for Animal Experimentation at National Research Institute of Fisheries Science (NRIFS), Fisheries Research Agency. All experimental protocols were approved by the Animal Research Committee of NRIFS.

### Sample collection, DNA extraction, and sequencing

For *de novo* genome assembly, a female Pacific bluefin tuna, an F1 specimen (parents from the wild) was collected in 2015. The phenotypic sex was determined by the histological sectioning of the gonad. DNA was extracted from blood using the standard phenol-chloroform protocol. Paired-end (PE) and mate-pair (MP) libraries were prepared for an Illumina platform using TruSeq Nano DNA LT Kit (Illumina) and Nextera Mate Pair Sample Preparation Kit (Illumina), respectively. Each library was shared to obtain an approximate insert size of 240 bp, 360 bp, 480 bp, and 720 bp for the PE libraries. The MP libraries had insert sizes of 3–5 kb, 10 kb, 20 kb, and 40 kb. The libraries were sequenced by NextSeq. 500. A SMRT-bell template library was prepared following the manufacturer’s protocol and was sequenced through a Pacific Biosciences (PacBio) platform (P6C4 chemistry).

For whole genome resequencing, a total of 31 individuals (male = 15, female = 16) were collected off the Nansei Islands, Japan, in 2015. After DNA extraction using Promega Maxwell RSC, PE libraries were prepared with insert sizes of 350–450 bp before sequencing by NextSeq. 500. An additional 56 individuals (male = 24, female = 32) were collected from off the Nansei Islands, while a further 59 individuals (male = 32, female = 27) were collected from the southern region of the Sea of Japan. These individuals were used for sex identification using PCR assays. Sex was identified by visual observation of the gonads.

### *de novo* genome assembly

Hybrid assembly with a combination of Illumina sequence data (PE and MP reads) and PacBio long molecule sequence reads was applied to construct the best possible assembly results, based on Chakraborty *et al*.^35^. PacBio subreads were filtered using SMRT Analysis 2.3.0 (minimum subread length 50, minimum polymerase read quality 75, minimum polymerase read length 50). Using CLC Genomics Workbench v. 9.5.2, raw Illumina reads were first quality filtered and short reads < 50 bp were discarded, and then adapter and duplicate reads were trimmed. A total of 240 bp Illumina reads were merged using CLC workbench. The filtered Illumina data were down-sampled randomly to achieve approximately ×65 coverage of the genome for De Bruijn graph assembly using Platanus v. 1.2.4^54^. Subsequently, the PacBio data were aligned to the De Bruijn graph assembly using DBG2OLC^55^ to produce a “backbone_raw.fasta” (k = 17, AdaptiveTh = 0.0001, KmerCovTh = 2, MinOverlap = 20, RemoveChimera = 1). The backbone_raw.fasta was used to generate consensus using the programs BLASR v.5.3, commit ec4144f^56^ and PBDagCon (downloaded from https://github.com/PacificBiosciences/pbdagcon). CANU v. 1.4^57^ was used for the raw PacBio reads (genomeSize = 850 m, corMinCoverage = 0, errorRate = 0.035), followed by a polishing process using Pilon v. 1.21^58^, and aligning PE, MP, and merged-240 bp Illumina reads to the PacBio assembly. QuickMerge v. 0.2^35^ was used to merge polished CANU and DBG2OLC assemblies by finding the best unique alignment between the assemblies using the program MUMmer v. 3.23^59^. Scaffolding was subsequently performed by aligning MP Illumina reads to QuickMerge processed consensus using BESST v. 2.2.5^60^. Finally, to generate the final consensus with gap correction using PE Illumina data and BESST consensus, GMcloser v. 1.6^61^ was used with Nucmer aligner^59^ (-l = 140, -i = 480, -d = 240, -c = TRUE). The completeness assessment was performed on the draft genomes using gVolante^62^ by analysing it with the CEGMA program^63^ and selecting the Core Vertebrate Genes (CVG)^64^ as an ortholog set. Benchmarking Universal Single-Copy Orthologs (BUSCO)^65^ was also performed using the odb9 actinopterygii ortholog dataset.

### Whole genome resequencing and variation calling

Whole genome resequencing data from 31 individuals were trimmed using Trimmomatic v. 0.36^66^ (CROP:145 LEADING:30 TRAILING:20 SLIDINGWINDOW:4:20 MINLEN:50). Duplicated reads were removed using ParDRe^67^. The filtered reads were mapped to a new draft genome sequence constructed using BWA-MEM v. 0.7.12^68^. Single nucleotide polymorphisms (SNPs) were identified using Unifiedgenotyper in Genome Analysis Toolkit (GATK) v. 3.6^69–71^ with no filtration. This approach reduces a large number of SNPs, as described in Star *et al*.^27^. Only biallelic SNPs were used for the subsequent analyses. Exact *p*-values using Fisher’s exact test in the 2-by-3 table of genotypes were calculated with PLINK v. 1.90b4.2^72^ (-fisher -model -hwe 0.0001). Haploview v. 4.2^73^ was used to find linkage disequilibrium (LD) blocks. Haplotypecaller in GATK was used to distinguish further variants for SNPs and insertion/deletion (indel) sites.

### PCR assay and sex identification

Three pairs of primers were manually designed according to unique sequences with strict sex-specific segregation (Table 1). An additional primer pair for amplifying a segment of mtDNA (the NADH dehydrogenase subunit 4 (*ND4*) gene) was prepared as an internal positive control. PCR amplification was conducted using a Takara PrimeSTAR GXL kit. The following PCR conditions were used, followed by 35 cycles: 10-s denaturation at 98 °C, 15 s annealing at 60 °C, 15 s extension at 68 °C; 0.5 μM final concentration for each sex-specific primer, and 0.06 μM for *ND4*; 0.5 ng/μL final concentration for the template; total volume 20 μL. PCR products were observed through electrophoresis or the MultiNA microchip electrophoresis system (Shimadzu).

### Gene annotation

The Fgenesh^74^ gene-finder was used to predict the gene structure using the *Oryzias latipes* gene model. Each predicted gene was translated to amino acid sequences. Functional annotation was carried out using BLASTP (E-value < 10^−15^) against the protein sequences of *Oryzias latipes* from the Ensembl database (release 90). Gene ontology descriptions were obtained from Ensembl to estimate the function of predicted genes. In addition, previously known SD genes, or genes associated with sex differentiation reported in other organisms, were located on the tuna draft genome using exonerate-2.2.0 (–model coding2genome, –bestn). These gene sequences were obtained from the NCBI and Ensemble (release 90) database.

## Supplementary information

Dataset1

## Data Availability

The datasets generated during the current study were deposited in the DNA DataBank of Japan (Accession No. DDBJ: DRA008331 for resequencing data and Accession No. DDBJ: BKCK01000001-BKCK01000444 for draft genome).
